# Demographic and Clinicopathological Spectrum of Gallbladder Carcinoma in a Tertiary Care Centre in Jharkhand: A Retrospective Study

**DOI:** 10.7759/cureus.63286

**Published:** 2024-06-27

**Authors:** Swati Priya, Manoj K Paswan, Arvind Kumar, Saurav Banerjee, Deepali Tirkey

**Affiliations:** 1 Pathology, Rajendra Institute of Medical Sciences, Ranchi, IND

**Keywords:** early-stage gallbladder carcinoma, gallbladder carcinoma in jharkhand, cholelithiasis in gallbladder carcinoma, gallbladder histopathology, incidental gallbladder carcinoma

## Abstract

Introduction

Gallbladder carcinoma is a rare but aggressive cancer of adults that affects females more than males. Its occurrence is more common in the regions of South America and Asia. Chronic inflammation and cholelithiasis are frequently associated risk factors of gallbladder carcinoma. The incidental discovery of a gallbladder carcinoma during cholecystectomy, gross or microscopic examination of the unsuspected gallbladder specimens is termed incidental gallbladder carcinoma (IGBC). Considering the lack of extensive studies on gallbladder carcinoma in the Eastern region of India, especially in Jharkhand, this study has been done to present the demographic and clinicopathological characteristics of gallbladder carcinoma in this region.

Methods

A retrospective and descriptive study was done at Rajendra Institute of Medical Sciences (RIMS), Ranchi, a tertiary care center in Jharkhand. The study sample comprised 2386 gall bladder cases received in the Department of Pathology over five years, from December 2018 to December 2023.

Results

Of 2368 specimens, 25 cases (n=25) were reported as primary gallbladder carcinoma. The female-to-male ratio was 4:1. Pain was the most common complaint by the patients. Of the 25 cases, 12 were suspected intra-operatively or diagnosed microscopically (IGBC). Most showed a mass at the neck. In six cases, no gross mass/lesion was seen. Cholelithiasis is present in 19/25 cases. Most cases showed adenocarcinoma (not otherwise specified). Out of the adenocarcinoma cases, most were well differentiated. At the time of diagnosis, most were at the pT2 stage. Twelve cases of IGBC were found. Eight out of 12 IGBC were early-stage carcinoma when diagnosed.

Conclusion

Twenty-five cases of gallbladder carcinoma were diagnosed in the last five years in our center, with 19 (76%) of them associated with cholelithiasis. Twelve (48%) of the cases were incidentally diagnosed either preoperatively or during gross/microscopic examination, and eight (66%) of those were discovered early, out of which five (62.5%) were observed to be in the T1b stage. At this stage, there is a diversion from the general surgical management of gallbladder carcinoma for a better prognosis. This underscores the significance of routine histopathological examination of gallbladder specimens, even if there is no preoperative suspicion of gallbladder carcinoma.

## Introduction

The most widespread malignant biliary tract tumor is gall bladder carcinoma [1]. Although uncommon, gall bladder carcinoma shows a higher mortality rate; its occurrence is distributed in an extended area worldwide [2]. It is observed that a large number of instances of gall bladder carcinoma happen in the regions of South America and Asia [3]. Out of these regions, the highest incidence of gall bladder carcinoma has been described in women of Delhi (21.5/100,000), South Karachi, Pakistan (13.8/100,000), and Quito, Ecuador (12.9/100,000) [1]. Jammu and Kashmir, Punjab, Haryana, Himachal Pradesh, Uttarakhand, Uttar Pradesh, Bihar, West Bengal, Assam, and Manipur are the Indian states that exhibit a raised risk of gall bladder carcinoma [4]. Gallbladder cancer is an ailment that occurs in adults and much more frequently in females than males [5]. Patients usually present with vague clinical features that mimic the symptoms of chronic cholecystitis, that is, nausea, pain in the right upper quadrant of the abdomen, vomiting, features of obstructive jaundice, or sometimes no symptoms at all, which ultimately leads to late diagnosis [6,7]. The incidental discovery of a gallbladder carcinoma during cholecystectomy, gross or microscopic examination of the unsuspected gallbladder specimens is termed incidental gallbladder carcinoma (IGBC) [8].

Chronic cholecystitis and gallbladder stones are the most frequently associated risk factors with the development of gallbladder cancer. It has been observed that chronic inflammation poses a critical risk in the development of gall bladder carcinoma. Also, gall bladder stone size, its type, the duration of the inflamed state, and the load of the symptoms strongly correlate with the occurrence of gall bladder cancer [6,9]. Some patients afflicted with undiagnosed gallbladder carcinoma may present with a mass, but in most cases, both radiological scans and gross examination are unable to ascertain its presence [9]. These cases show gallbladder wall thickening and mucosal ulceration, thus culminating in a late diagnosis and a poorer outcome [10,11]. Owing to the hostile character of the gall bladder tumor and the complex anatomy of its adjoining structures, there is a rapid malignant extension in the contiguous region, which in turn is responsible for a dismal 5% overall survival rate in gall bladder carcinoma with a median survival time of approximately six months [12,13]. However, a few cases in which incidental gallbladder carcinoma is observed to be at an early stage show a five-year survival rate of 80% [14]. This study was done after observing a lack of extensive studies on gallbladder carcinoma in the Eastern region of India, especially in Jharkhand.

Aim

In this study, we aim to present the demographic and clinicopathological characteristics of gallbladder carcinoma, including the incidence rate of incidental gallbladder carcinoma in a tertiary care center of Jharkhand, which belongs to a high-risk zone for developing gallbladder carcinoma.

## Materials and methods

This study was done at Rajendra Institute of Medical Sciences (RIMS), Ranchi, a tertiary care center in Jharkhand, India, after approval from the Institutional Ethics Committee, No. 144, dated 23/04/24. It was a retrospective, descriptive study. The study sample comprised 2386 gall bladder cases received in the Department of Pathology over five years, from December 2018 to December 2023.

Inclusion and exclusion criteria

All gall bladder specimens received in the histopathology section from December 2018 to December 2023 were reported as gall bladder carcinoma of any type. The cases reported as benign gallbladder lesions and the ones showing mild dysplastic changes were excluded. The cases whose relevant clinical data was missing were also excluded.

The gallbladder specimens received in our tertiary center were screened for cases reported as gallbladder carcinoma. The gallbladder carcinoma cases were further scrutinized to assess their demographic profile, relevant clinical history (presenting complaints, preoperative diagnosis, intraoperative findings), grossing details, and histopathological details. Additional focus was provided to analyze the cases of incidental gallbladder carcinoma. The data was manually retrieved from the histopathology registers kept in the pathology department. The staging of the tumours was done according to the American Joint Committee on Cancer (AJCC) Seventh Edition. The blocks of the cases that were available in the department were recut and stained with hematoxylin and eosin stain. The slides were then viewed under the microscope, and appropriate micro-pictographs were clicked. 

Statistical analysis

All the detailed gross, microscopic studies, clinical history, radiological, and intraoperative findings noted in the registers were studied. The data was entered into a Microsoft Excel sheet (Microsoft, Redmond, Washington) and analyzed. Association of cholelithiasis with gallbladder carcinoma was done as well as the number and size of the stones received in such cases were also taken into account. The incidental gallbladder carcinoma were analyzed separately. Lastly, the presentation of data was done in a tabular form using tables, pie charts, and bar diagrams.

## Results

**Figure 1:**
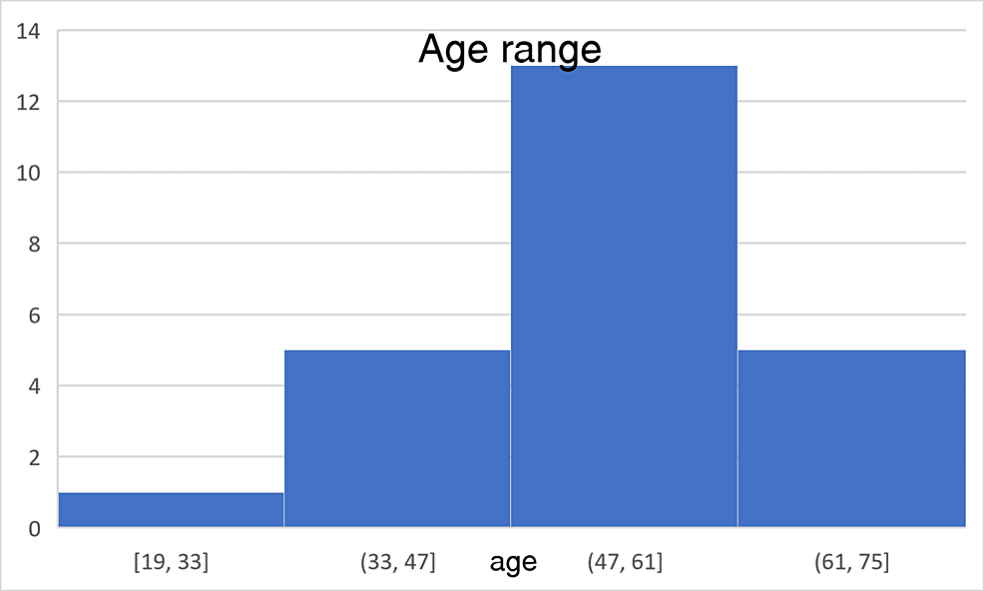
A histogram showing the age range of the patients diagnosed with gallbladder carcinoma The majority (13) of patients' ages were between 47 and 61 years

**Figure 2:**
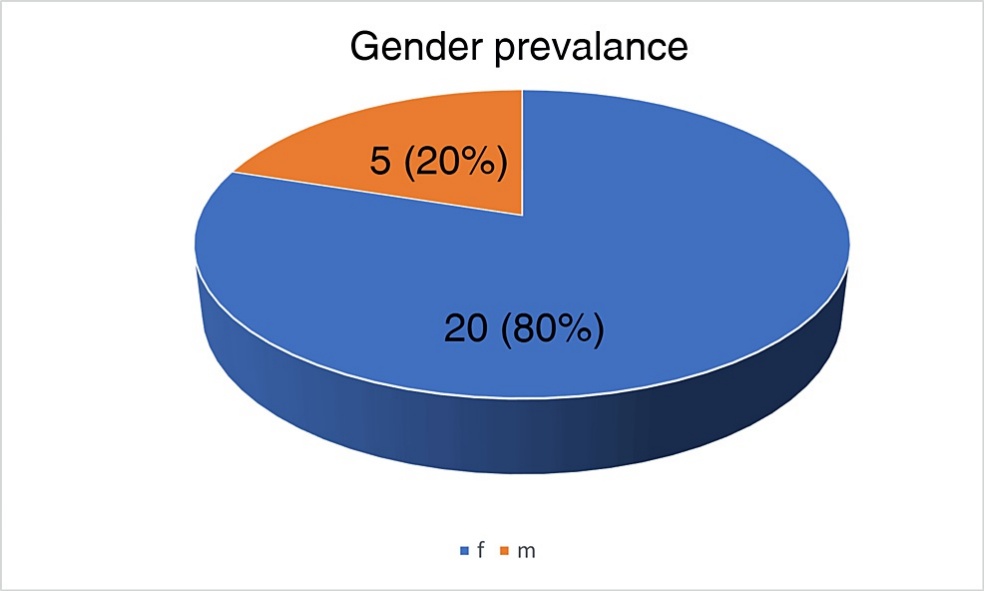
A pie-chart showing the frequency distribution of gallbladder carcinoma among the gender of the participants 80% represent females, 20% represent males. The majority of patients diagnosed with gallbladder carcinoma were females

The histopathology section of the Department of Pathology, RIMS, Ranchi, received 2368 gallbladder specimens between December 2018 and December 2023. Of these specimens, 25 cases (n=25) were reported as primary gallbladder carcinoma. The age of the patients varied from 19 years to 75 years, with a peak in the 50th decade, as shown in Figure 1. There were 20 females and five males with a female-to-male ratio of 4:1, shown in the form of a pie chart in Figure 2. The mean age for malignancy was 53.2 years, 52.1 years in females, and 57.6 years in males. The demographic details are given in Table 1. 

**Table 1:** Demographic details of the patients who were diagnosed with gallbladder carcinoma

Sl no. (n=25)	Age	Sex
1	40	F
2	66	M
3	53	F
4	48	M
5	54	M
6	43	F
7	52	F
8	64	F
9	58	F
10	68	F
11	64	M
12	60	F
13	56	M
14	35	F
15	69	F
16	55	F
17	61	F
18	19	F
19	60	F
20	60	F
21	45	F
22	58	F
23	38	F
24	52	F
25	53	F

**Figure 3:**
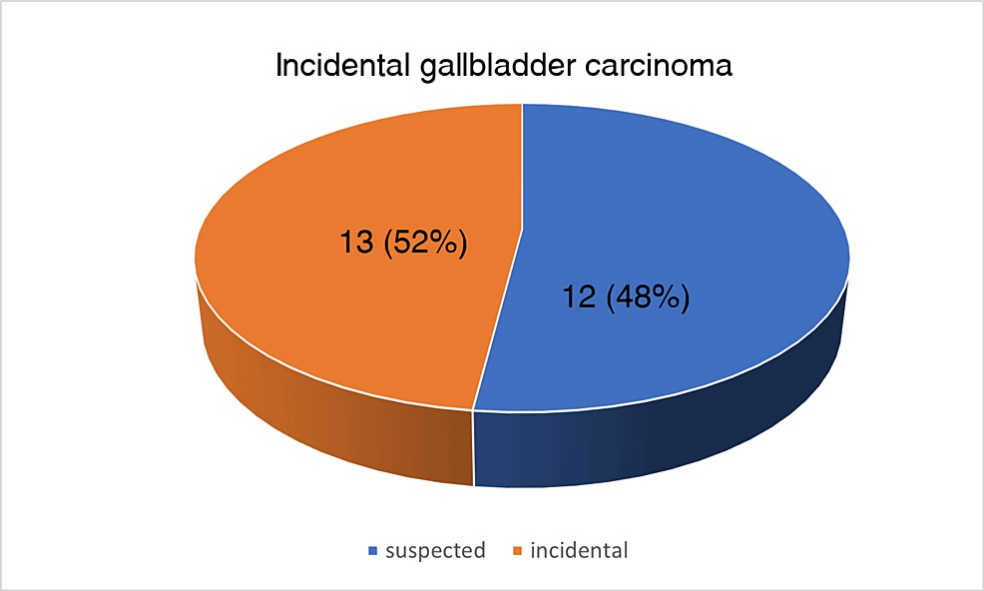
A pie chart showing percentage of incidentally diagnosed gallbladder carcinoma (either intraoperatively or after gross/microscopic examination)

**Figure 4:**
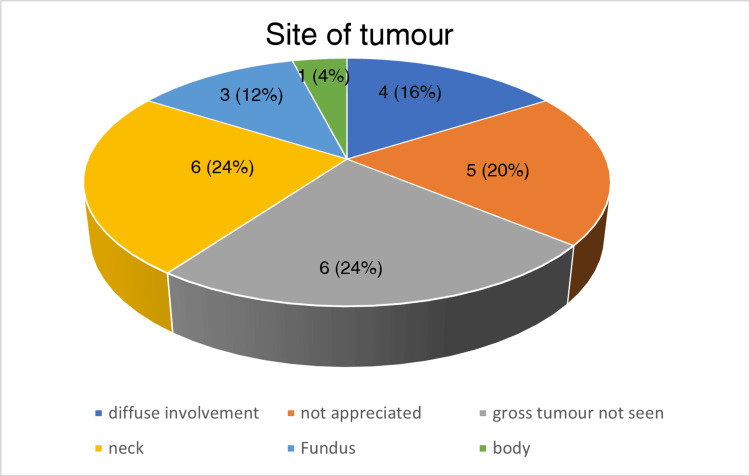
A pie chart showing the tumor site as observed during grossing of the gallbladder carcinoma specimen In six (24%) cases, the site of the tumor was in the neck region of the gallbladder, in four (20%) there was diffuse involvement, in three (12%) the site of the tumor was seen in the fundus region of the gallbladder, in one (4%) the site of the tumor is the body of the gallbladder. In another six cases (24%) no gross tumor was seen and in five cases (20%) the site of the tumor could not be appreciated since the specimens were sent in pieces to the pathology department.

**Figure 5:**
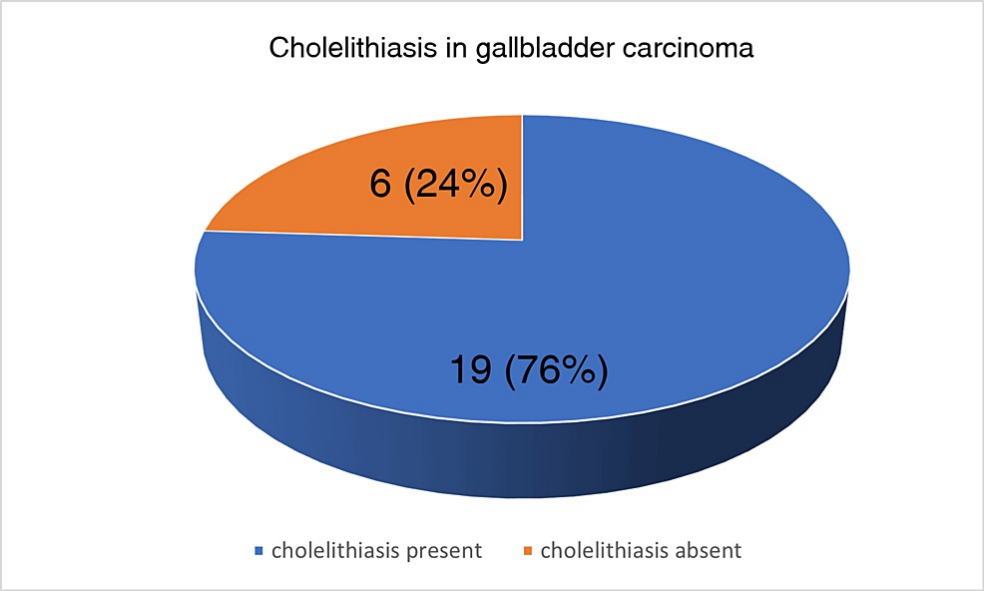
A pie chart showing the number of gallbladder carcinoma cases associated with cholelithiasis In 76% of the cases, cholelithiasis was present, in 24% of the cases it was absent. The majority (76%) of the cases of gallbladder carcinoma were associated with cholelithiasis.

Pain was the most common complaint by the patients, seen in 18/25 cases. Right hypochondriac region pain was seen in 13/25 cases; in five cases, the patients complained of pain in the epigastric region. In 10 of these cases, pain was associated with nausea/vomiting. In 6/25 cases, no specific complaints were present. Of the 25 cases, 13 were suspected of malignancy after an abdominal ultrasound and abdominal CT scan, and 12 were suspected intra-operatively or diagnosed microscopically (incidental gall bladder carcinoma), as depicted in Figure 3. On gross examination of the cut open section, the site of the tumor could not be assessed in five cases since the gall bladder specimens were sent in pieces, diffuse involvement was seen in four, six showed a mass at the neck region, three at the fundus region, and one at the body region. In six cases, no gross mass/lesion was seen. The site of the tumor observed during grossing is depicted in the form of a pie chart in Figure 4. The thickness of the gallbladder wall was more than or equal to 0.4 cm in all the cases sent in an intact form. Cholelithiasis is present in 19/25 cases, as depicted in Figure 5. In four such cases, the gall bladder was sent in pieces, and the details of the type of stones found were not given. Twelve cases showed an association with cholesterol stones, and three showed pigment stones. In 2/15 cases, a single stone was found, and in 13/15 cases, multiple stones were observed. In 3/15 cases, the size of the gall stones was less than 1 cm; in 9/15 cases, the stone size lies between 1 cm and 2 cm; and in 3/15 cases, it is more than 2 cm. These clinical details are presented in a tabular form in Table 2.

**Table 2:** Clinical details of the patients who were diagnosed with gallbladder carcinoma and grossing details of the specimen received

Sl no. (n=25)	Clinical presentation	Site of gross tumour	Cholelithiasis	Nature and number of stones	Suspected/incidental gallbladder carcinoma	Size of stone
1	Pain in the right upper quadrant	Not appreciated	Present	No stones received	Suspected	-
2	Pain in the epigastric region, jaundice	Fundus	Present	Yellowish-green stones, 3	Suspected	1.2 cm
3	No specific symptom	Fundus	Absent	No stones	Suspected	-
4	Pain in the right upper quadrant	Neck	Present	Black stones, 2	Incidental	1.5 cm
5	No specific symptoms	Gross tumor not seen	Present	No stones received	Incidental	-
6	Pain in the epigastric region, jaundice	Diffuse involvement	Absent	No stones	Suspected	-
7	Pain in the right upper quadrant, jaundice, nausea	Neck	Absent	No stones	Suspected	-
8	Nausea	Gross tumor not seen	Present	Yellowish-green stones, 2	Incidental	2 cm
9	No specific symptoms	Not appreciated	Absent	No stones	Suspected	-
10	Pain in the right upper quadrant, nausea	Not appreciated	Present	No stones received	Suspected	-
11	Pain in the epigastric region, jaundice	Gross tumor not seen	Present	Yellowish-white stones, multiple	Incidental	0.8 cm
12	Pain in the right upper quadrant	Diffuse involvement	Present	Yellowish white stones, 1	Incidental	3.5cm
13	Pain in the right upper quadrant, jaundice, vomiting	Fundus	Absent	Not stones	Suspected	-
14	Pain in the right upper quadrant, jaundice	Diffuse involvement	Present	Yellowish white stones, multiple	Suspected	1.5 cm
15	Pain in the right upper quadrant, jaundice, nausea	Neck	Present	Yellow stone, 1	Suspected	3 cm
16	Pain in the epigastric region	Diffuse involvement	Present	Yellowish white stones, multiple	Incidental	1 cm
17	Pain in the right upper quadrant, jaundice, nausea	Neck	Present	Yellowish white stone, multiple	Suspected	1.2 cm
18	No specific symptoms	Not appreciated	Absent	No stones	Incidental	-
19	Pain in the right upper quadrant	Gross tumor not seen	Present	Yellowish white stones, multiple	Incidental	0.4 cm
20	Pain in the epigastric region, nausea	Body	Present	Greyish black stones, 2	Suspected	2.6 cm
21	Pain in the right upper quadrant	Neck	Present	Greenish-yellow stones, multiple	Incidental	1.5 cm
22	Pain in the right upper quadrant	Not appreciated	Present	Not received	Suspected	-
23	No specific symptoms	Neck	Present	Greyish black stone, multiple	Incidental	0.5 cm
24	No specific symptoms	Gross tumor not seen	Present	Yellowish-white stone, multiple	Incidental	1 cm
25	Pain in the right upper quadrant	Gross tumor not seen	Present	Yellowish white stones, multiple	Incidental	1 cm

**Figure 6:**
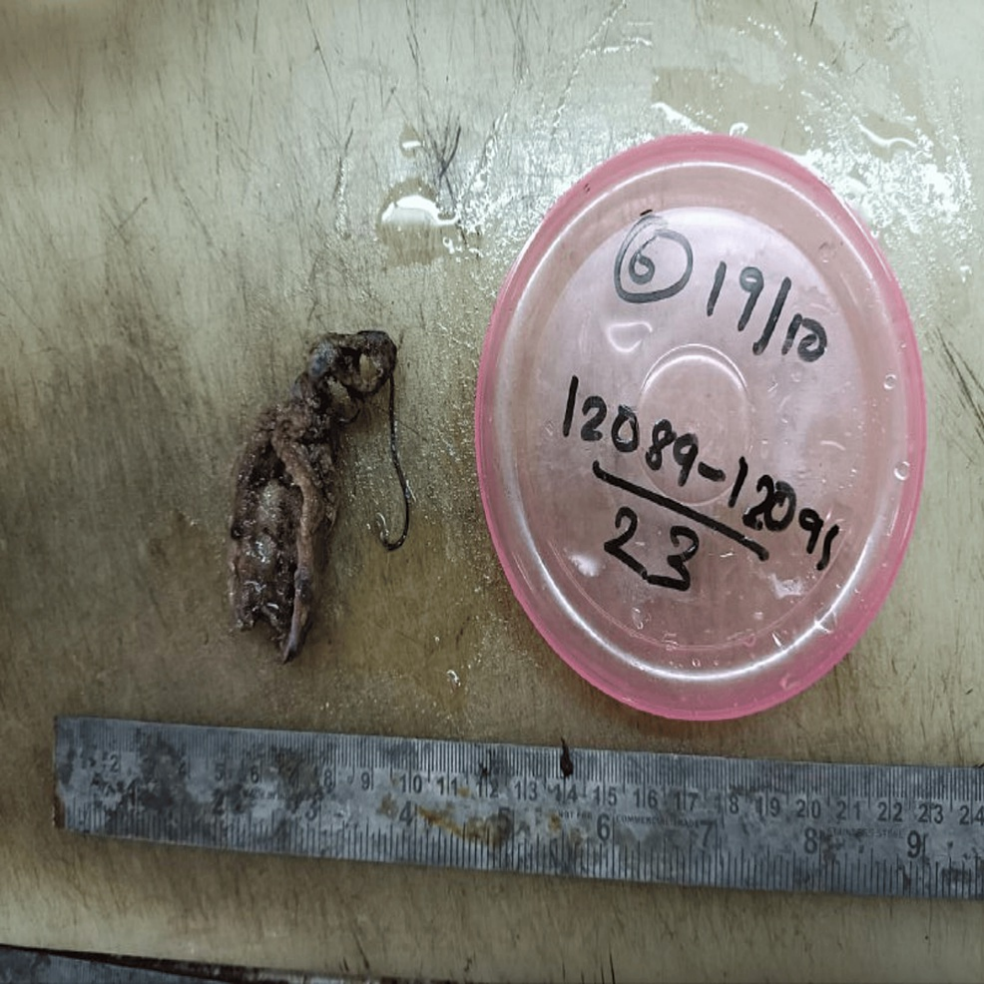
A gross specimen of an already cut-open gallbladder showing an exophytic mass in the body region of the gallbladder. Two greyish-black stones were sent separately.

**Figure 7:**
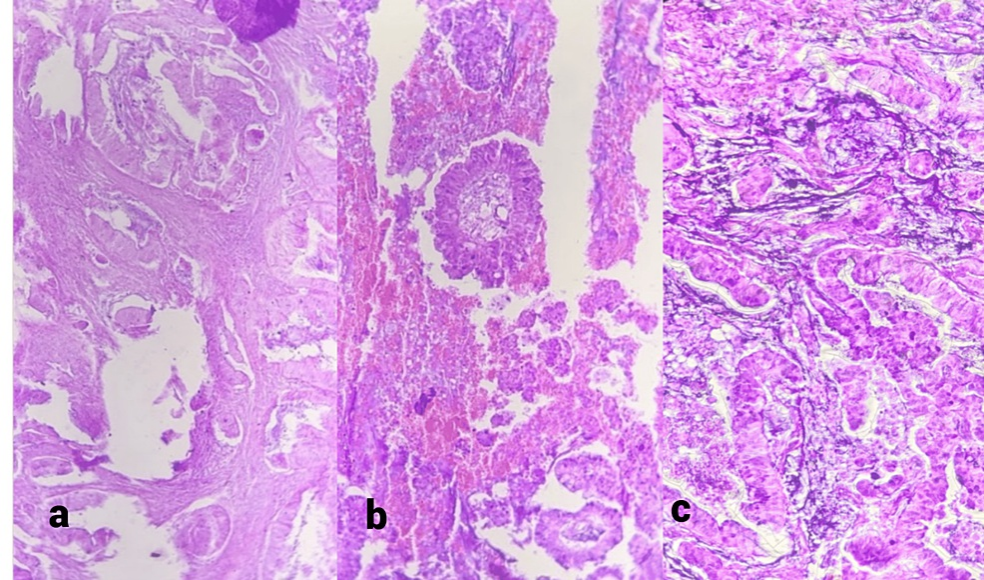
Papillary variant of adenocarcinoma a) 10x, showing papillary architecture of the tumor, b) 20x, few papillae are seen with fibrovascular cores, c) 40x, showing back-to-back arranged papillae lined by malignant epithelial cells showing stratification

**Figure 8:**
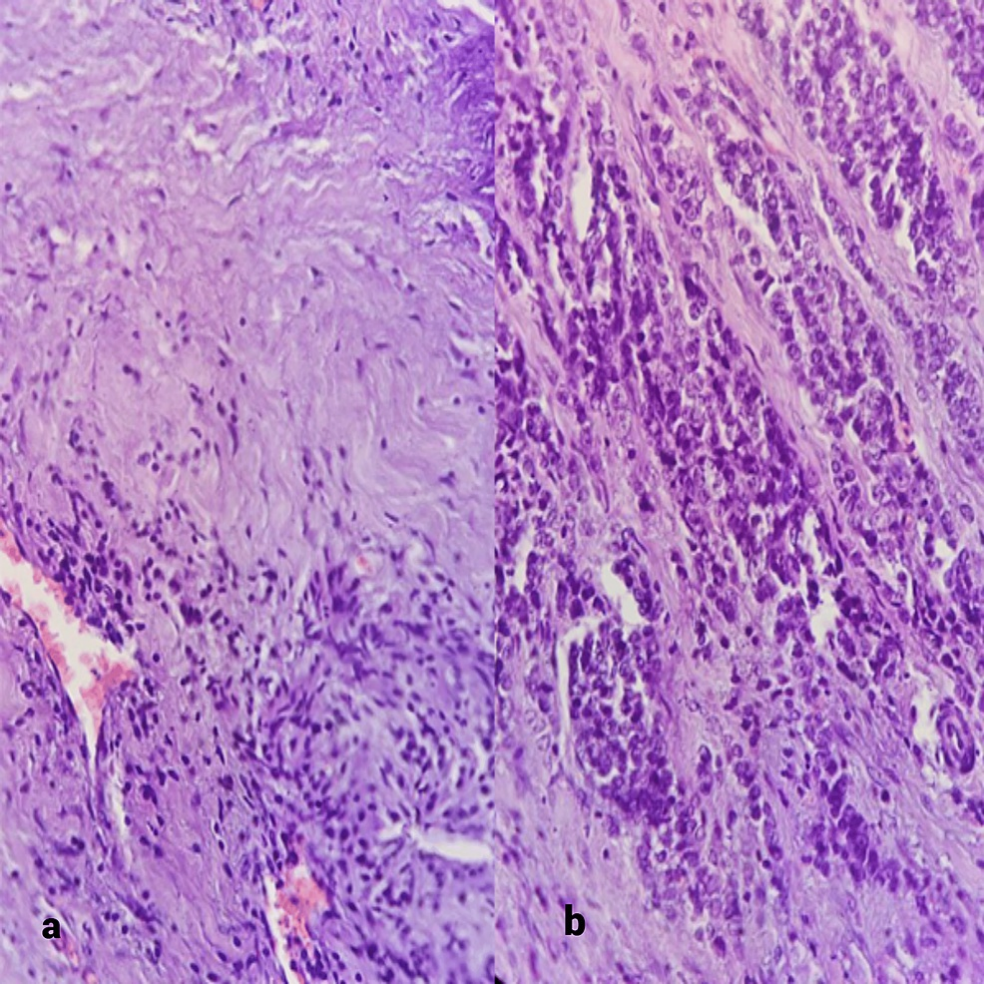
A mucinous variant of gallbladder carcinoma a) 20x, tumor cells seen in pools of mucin, b) 40x, tumor cells arranged in cords and trabeculae, some are attempting to form glandular structures, in the background of mucin

**Figure 9:**
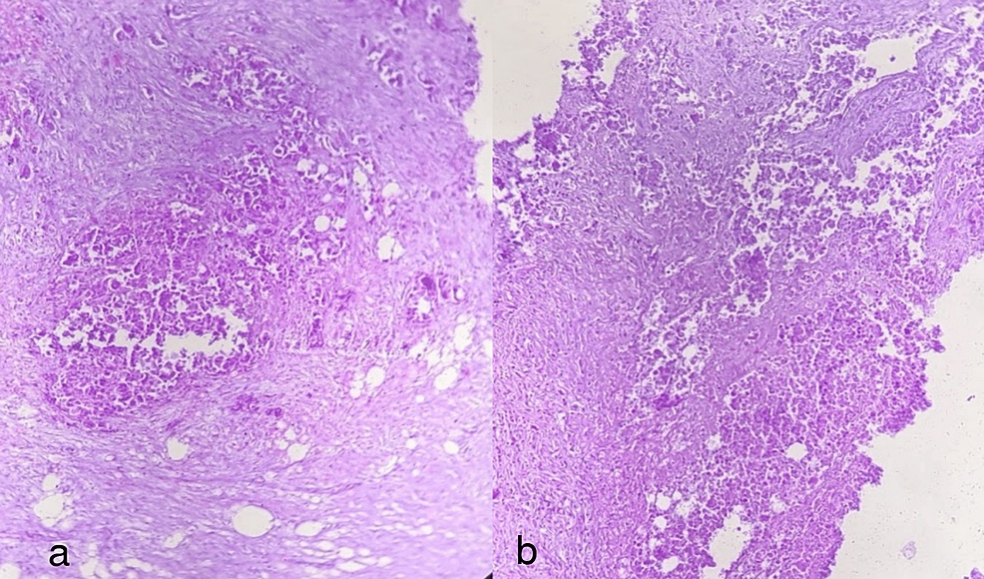
Poorly differentiated adenocarcinoma (not otherwise specified), a and b) 20x, these images show few tumor cells forming small glands with poorly formed lumen. Tumor cells are also arranged in the cell nests and lie singly infiltrating the smooth muscle layer of the gallbladder

**Figure 10:**
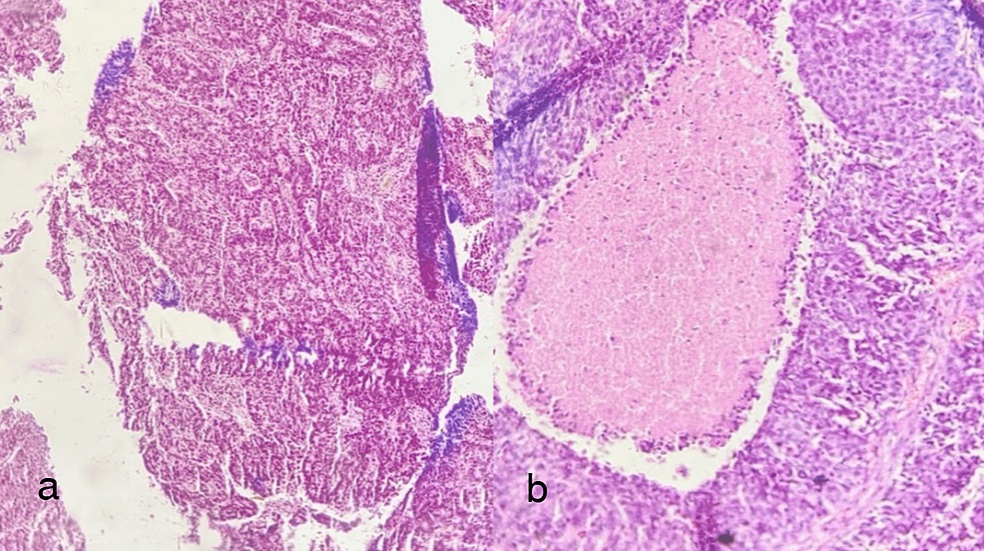
Moderately differentiated adenocarcinoma (Not otherwise specified) a) 10x, well-formed glands admixed with small glands visible which have abortive lumens and solid cell nests, b) A focus of comedonecrosis seen

Most cases showed adenocarcinoma not otherwise specified (NOS), with five cases showing a papillary variant of the adenocarcinoma and one case each of adenocarcinoma with squamous metaplasia, a mucinous variant of adenocarcinoma, and squamous cell carcinoma. Out of the adenocarcinoma (NOS), eight were well differentiated, and five were moderately or poorly differentiated. The histomorphological details of the different types of gallbladder carcinoma have been depicted and discussed in Figures 6-11. On observing the Tumour, Node, Metastasis (TNM) staging of the gallbladder carcinoma at the time of diagnosis, 9/25 cases were at the pT1 stage, 10 were at the pT2 stage, five were at the pT3 stage, and one was at the pTis stage. The TNM staging was done according to AJCC 7th edition. The histopathological spectrum of gallbladder carcinoma with their staging is given in Table 3.

**Table 3:** Histopathological details and staging of the gallbladder carcinoma TNM - Tumour, Node, Metastasis

Sl no. (n=25)	Histopathological spectrum	TNM staging
1	Moderately differentiated adenocarcinoma	T1bNMx
2	Well-differentiated adenocarcinoma	T2N1Mx
3	Well-differentiated adenocarcinoma	TisNxMx
4	Moderately differentiated adenocarcinoma	T1aNxMx
5	Well-differentiated adenocarcinoma	T1bNxMx
6	Poorly differentiated adenocarcinoma	T3NxMx
7	Adenocarcinoma with squamous metaplasia	T3NxMx
8	Adenocarcinoma papillary variant	T2NxMx
9	Adenocarcinoma papillary variant	T2NxMx
10	Poorly differentiated adenocarcinoma	T2NxMx
11	Mucinous adenocarcinoma moderately differentiated	T2NxMx
12	Adenocarcinoma papillary variant	T1aNxMx
13	Well-differentiated adenocarcinoma	T3NxMx
14	Well-differentiated adenocarcinoma	T3NxM1
15	Well-differentiated adenocarcinoma	T2NxMx
16	Well-differentiated adenocarcinoma	T1bNxMx
17	Adenocarcinoma papillary variant	T3N1Mx
18	Poorly differentiated adenocarcinoma	T2NxMx
19	Moderately differentiated adenocarcinoma	T1bNxMx
20	Well-differentiated squamous cell carcinoma	T2N1M1
21	Well differentiates adenocarcinoma	T2N1Mx
22	Poorly differentiated adenocarcinoma	T2NxMx
23	Adenocarcinoma papillary variant	T1aNxMx
24	Moderately differentiated adenocarcinoma	T1bNxMx
25	Moderately differentiated adenocarcinoma	T1bNxMx

Twelve cases of incidental gallbladder carcinoma were found which means they were either discovered intraoperatively, by gross examination, or only after microscopic examination of the gall bladder tissue. Eight out of 12 IGBC were early-stage carcinoma when diagnosed. The demographic clinicopathological details of the IGBCs are given in Table 4.

**Table 4:** Demographic and histopathological details of incidental gallbladder carcinoma GB - gallbladder; NOS - not otherwise specified

Sl no. (n=25)	Age	Sex	Pre-operative diagnosis	Intraoperative details	Gross findings	Histopathological diagnosis	TNM stage
1	48	M	GB empyema	No specific details given	A mass was found in the neck region of the gallbladder	Moderately differentiated adenocarcinoma (NOS)	pT1a
2	54	M	Choledocholithiasis	Necrotic areas are seen, Ca GB	No gross tumour	Well-differentiated adenocarcinoma (NOS)	pT1b
3	64	F	Cholelithiasis	GB perforation	No gross tumour	Adenocarcinoma papillary variant	pT2
4	64	M	Choledochal cyst	No specific details given	No gross tumour, on cut open section - copious mucinous fluid	Adenocarcinoma mucinous variant moderately differentiated	pT2
5	60	F	Cholelithiasis	No specific details given	Diffuse involvement of the gall bladder by a fungating growth	Adenocarcinoma papillary variant	pT1a
6	55	F	Cholelithiasis	Distended GB with multiple stones, the wall thickened and fibrosed	Diffuse involvement of the gallbladder lumen	Well-differentiated adenocarcinoma (NOS)	pT1b
7	19	F	GB empyema	GB necrosed	Not appreciated (GB received in pieces)	Poorly differentiated adenocarcinoma (NOS)	pT2
8	60	F	Cholelithiasis	Sludge present	No gross tumour	Moderately differentiated adenocarcinoma (NOS)	pT1b
9	45	F	Cholelithiasis	GB wall thickened and fibrotic, one enlarged periportal lymph node discovered	A mass was found in the neck region of the gallbladder	Well-differentiated adenocarcinoma (NOS)	T2N1Mx

## Discussion

Gallbladder carcinoma is a rare but aggressive cancer usually diagnosed at later stages with a dismal overall survival rate. [1,2,12,13]. If detected early, the prognosis is better, but the diagnosis is often masked by non-specific symptoms [6,7,14]. The incidence rate of gallbladder cancer in our center was observed to be 25/2386 (1.04%) within the last five years. The female-to-male ratio seen in our study is 4:1. A diverse series of Indian studies have shown that the male-to-female ratio is from 3:1 to 4.5:1 [15]. In recent years, there has been a rise in the incidence of gallbladder carcinoma among females. The average age-adjusted rate of gallbladder carcinoma in women has grown from 6.2/100,000 in 2001-2004 to 10.4/100,000 in 2012-2014 [16]. The average age at diagnosis of gallbladder carcinoma in India was 51±11 years, considerably younger in contrast to 71.2±12.5 years in the USA and Western European countries [14,17,18]. There have been several studies in which the mean age of Indian gallbladder carcinoma patients at the time of diagnosis was 50 to 55 years [19]. In our study, the mean age for malignancy was 53.2 years, in females, 52.1 years, and in males 57.6 years. Thirteen (52%) of the patients presented with pain in the right hypochondriac region and five (20%) in the epigastric region. Of the cases presenting with pain, 55.5% were having complaints of jaundice and nausea/vomiting. In 24% of the cases, no significant complaints were present. Gallbladder stones are a chief factor for the development of gallbladder carcinoma, showing 8.3 times more risk than the unaffected population. The chronic irritation of the gallbladder mucosa due to the development of gallbladder stones is one of the important steps in the transformation to carcinoma [2,6,9]. In 70-90% of patients with gallbladder carcinoma, gallstones were present, as reported by various studies in India [14,20,21]. This study found that 19 (76%) cases were associated with cholelithiasis, similar to the trend shown in those studies. Furthermore, in this study, 13 (86%) of the gallbladder with cholelithiasis in which gall stones were received had more than one stone, 12 (63%) were cholesterol stones, and nine (60%) were between 1 cm and 2 cm in size. The findings related to the number and nature of stones are similar to those of a study by Narang et al. In that study, the gallbladder carcinoma cases had stones of more than 2 cm in size, but in ours, the majority lie between 1 cm and 2 cm [22]. In six (24%) cases, a gross tumor was found in the neck region. Three (12%) of the cases showed a gross tumor in the fundus of the gallbladder and one (4%) in the body of the gallbladder. In four (16%) of the cases, a diffuse gallbladder involvement is seen. In the rest of the intact gallbladder specimens, six (24%) of the cases did not show any gross tumor or suspicious lesion.

There is a lack of understanding of the pathogenesis of gallbladder carcinoma in India, even today. Several external and host factors cause epigenetic and genetic mutations leading to tumorigenesis. Any impediment in emptying of the gallbladder adds to the anatomically weak muscular structure of the gallbladder and favors the growth of bacteria. It also leads to the release of toxic metabolites, which ultimately cause chronic inflammation. Moreover, the presence of gallbladder stones and chronic cholecystitis severely impact the emptying of the gallbladder, thus perpetuating the molecular insult [19]. Various studies from India have shown a mutation of the p53 pathway in about 70% of the cases [23]. 

Various studies report the incidence of gallbladder carcinoma diagnosed during or after a laparoscopic cholecystectomy to be between 0.19-3.3% [24,25,26]. This study found that the incidence rate of incidental gallbladder carcinoma over the last five years was 12/2386 (0.5%) in our center. We observed in our study that a preoperative diagnosis of gallbladder carcinoma by using imaging techniques could not be done in 12 (48%) of the gallbladder carcinoma detected, stressing the pivotal role of histopathological examination in detecting gallbladder carcinoma. Although the five-year survival rate of gallbladder carcinoma is dismal, it is more than 80% in a handful of cases where early-stage malignancies are incidentally found during cholecystectomy in cholelithiasis [14]. Among all the gallbladder carcinoma cases in this study, 10 (40%) were at the pT2 stage, and five (20%) were at the pT3 stage. Eight out of 12 (66%) of incidental gallbladder carcinoma was diagnosed at an early stage in our study. Tantia et al. also found the majority of incidental carcinomas at an early stage [25]. Of those, five (62.5%) were at the pT1b stage. The cases where the stage at the time of diagnosis was higher than pT1a re-resection and re-exploration are suggested, given that metastatic dissemination is not seen [27]. We observed 24/25 cases of various subtypes of adenocarcinoma and a single case of squamous cell carcinoma, similar to many studies, including a study by Weinstein et al. [28].

The impact of the endemicity of *Salmonella typhi* and *Helicobacter *in a geographical region, especially in low socioeconomic setting, on the increased rate of gall bladder cancer is under study, hinting that there is a direct relationship between the chronic carrier state of these two microorganisms and gall bladder cancer incidence [29]. Given the raised incidence of gallbladder carcinoma in women, recent studies are underway to study the presence of estrogen and progesterone receptors on the gallbladder mucosa, which may promote gallbladder stasis and stone formation, which may increase the gallbladder mucosa's exposure time to bacterial and chemical toxins [30]. 

## Conclusions

In this study, we report 25/2386 cases of gallbladder carcinoma diagnosed in the last five years in our center with 19 (86%) of them associated with cholelithiasis. Twelve (48%) of the cases were incidentally diagnosed either preoperatively or during gross/ microscopic examination, and eight (66%) of those were discovered early, of which five (62.5%) were observed to be in the T1b stage. At this stage, there is a diversion from the general surgical management of gallbladder carcinoma for a better prognosis. This underscores the significance of routine histopathological examination of gallbladder specimens even if there is no preoperative suspicion of gallbladder carcinoma.

Since the incidence of gallbladder carcinoma is rare, the sample size of the cases is small. Additional studies such as correlation with chronic infections such as with *Salmonella typhi* and *Helicobacter pylori* could not be done. Moreover, immunohistochemical studies could not be done to assess the hormonal status of gallbladder carcinoma in females. As this geographical location is included in a high-risk zone for developing gallbladder cancers with the female age-adjusted incidence rate growing tremendously over the years, a collaborative study involving surgery, pathology, and microbiology departments of multiple medical institutions is required in the Eastern part of India in the future.

## References

[1] Randi G, Franceschi S, La Vecchia C (2006). Gallbladder cancer worldwide: geographical distribution and risk factors. Int J Cancer.

[2] Kanthan R, Senger JL, Ahmed S, Kanthan SC (2015). Gallbladder cancer in the 21st century. J Oncol.

[3] Roa I, Ibacache G, Muñoz S, de Aretxabala X (2014). Gallbladder cancer in Chile: pathologic characteristics of survival and prognostic factors: analysis of 1,366 cases. Am J Clin Pathol.

[4] Konstantinidis IT, Deshpande V, Genevay M (2009). Trends in presentation and survival for gallbladder cancer during a period of more than 4 decades: a single-institution experience. Arch Surg.

[5] Poudel R, Singh S, Basnet S (2015). Clinicopathological study of gall bladder cancer and its relationship with gall stones. J Soc Surg Nepal.

[6] Mukkamalla SKR, Kashyap S, Recio-Boiles A (2023). Gallbladder Cancer.

[7] Ben Kridis, Toumi N, Daoud J (2019). Gall Bladder Carcinoma: Clinical Presentations and Different Modalities of Treatment.

[8] Waghmare RS, Kamat RN (2014). Incidental gall bladder carcinoma in patients undergoing cholecystectomy: a report of 7 cases. J Assoc Physicians India.

[9] Roa I, Araya JC, Villaseca M, Roa J, de Aretxabala X, Ibacache G (1999). Gallbladder cancer in a high risk area: morphological features and spread patterns. Hepatogastroenterology.

[10] Cubertafond P, Gainant A, Cucchiaro G (1994). Surgical treatment of 724 carcinomas of the gallbladder. Results of the French Surgical Association Survey. Ann Surg.

[11] Sobin LH, Gospodarowicz MK, Wittekind C (2009). TNM Classification of Malignant Tumours. https://books.google.co.in/books?id=sUaevQ0I_8kC.

[12] Dutta U (2012). Gallbladder cancer: can newer insights improve the outcome?. J Gastroenterol Hepatol.

[13] Grobmyer SR, Lieberman MD, Daly JM (2004). Gallbladder cancer in the twentieth century: single institution's experience. World J Surg.

[14] Hundal R, Shaffer EA (2014). Gallbladder cancer: epidemiology and outcome. Clin Epidemiol.

[15] Roa I, Araya JC, Villaseca M, De Aretxabala X, Riedemann P, Endoh K, Roa J (1996). Preneoplastic lesions and gallbladder cancer: an estimate of the period required for progression. Gastroenterology.

[16] Phadke PR, Mhatre S, Budukh A (2019). Trends in gallbladder cancer incidence in the high- and low-risk regions of India. Ind J Med Paediatr Oncol.

[17] Alvi AR, Siddiqui NA, Zafar H (2011). Risk factors of gallbladder cancer in Karachi-a case-control study. World J Surg Oncol.

[18] Khan MR, Raza SA, Ahmad Z (2011). Gallbladder intestinal metaplasia in Pakistani patients with gallstones. Int J Surg.

[19] Dutta U, Bush N, Kalsi D, Popli P, Kapoor VK (2019). Epidemiology of gallbladder cancer in India. Chin Clin Oncol.

[20] Batra Y, Pal S, Dutta U (2005). Gallbladder cancer in India: a dismal picture. J Gastroenterol Hepatol.

[21] Shukla VK, Khandelwal C, Roy SK, Vaidya MP (1985). Primary carcinoma of the gall bladder: a review of a 16-year period at the University Hospital. J Surg Oncol.

[22] Narang Narang, Shveta & Goyal, Parul & Bal (2014). Gall stones size, number, biochemical analysis and lipidogram- an association with gall bladder cancer: a study of 200 cases. Int J Cancer Ther Oncol.

[23] Mishra PK, Jatawa SK, Raghuram GV (2009). Correlation of aberrant expression of p53, Rad50, and cyclin-E proteins with microsatellite instability in gallbladder adenocarcinomas. Genet Mol Res.

[24] Zhang WJ, Xu GF, Zou XP, Wang WB, Yu JC, Wu GZ, Lu CL (2009). Incidental gallbladder carcinoma diagnosed during or after laparoscopic cholecystectomy. World J Surg.

[25] Tantia O, Jain M, Khanna S, Sen B (2009). Incidental carcinoma gall bladder during laparoscopic cholecystectomy for symptomatic gall stone disease. Surg Endosc.

[26] Shrestha R, Tiwari M, Ranabhat SK Incidental gallbladder carcinoma: value of routine histological examination of cholecystectomy specimens. Nepal Med Coll J.

[27] Creasy JM, Goldman DA, Gonen M (2017). Predicting residual disease in incidental gallbladder cancer: risk stratification for modified treatment strategies. J Gastrointest Surg.

[28] Weinstein D, Herbert M, Bendet N, Sandbank J, Halevy A (2002). Incidental finding of gallbladder carcinoma. Isr Med Assoc J.

[29] Sharma J, Huda F, Naithani M (2022). Role of gut microbiome and enteric bacteria in gallbladder cancer. Immunology of the GI tract- Recent advances.

[30] Baskaran V, Vij U, Sahni P, Tandon RK, Nundy S (2005). Do the progesterone receptors have a role to play in gallbladder cancer?. Int J Gastrointest Cancer.

